# Inflammation and insulin profiles in men assigned to exercise vs. usual care for prostate cancer: results from the active surveillance exercise (ASX) randomized controlled trial

**DOI:** 10.1007/s10552-025-02102-3

**Published:** 2026-01-17

**Authors:** William A. Pace, Adam Olshen, Ye Wang, Stacey A. Kenfield, Erin L. Van Blarigan, Rebecca E. Graff, Janet Cowan, Lufan Wang, Imelda Tenggara, Karen Lopez, Katriel Encabo, Lee W. Jones, Neil Panchal, Matthew R. Cooperberg, Peter R. Carroll, Anthony Luke, Michael Pollak, June M. Chan

**Affiliations:** 1 Department of Urology, UCSF, San Francisco, CA USA; 2 Helen Diller Family Comprehensive Cancer Center, San Francisco, CA USA; 3 Department of Epidemiology and Biostatistics, UCSF, San Francisco, CA USA; 4 Department of Medicine and Oncology, McGill University, Montreal, Qc Canada; 5 Department of Medicine, Memorial Sloan Kettering Cancer Center, New York, NY USA; 6 Department of Orthopaedic Surgery, University of California San Francisco, San Francisco, CA USA

**Keywords:** Prostate cancer, Active surviellance, Exercise intervention, Inflammation, Insulinemia

## Abstract

**Objectives:**

Physical activity is associated with reduced risk of prostate cancer (PCa) progression and death; changes in insulin sensitivity and inflammation are potential mediating mechanisms. This study examined whether exercise after PCa diagnosis affects insulin-related and inflammatory biomarkers.

**Methods:**

The Active Surveillance Exercise (ASX) randomized controlled trial was assigned to men undergoing active surveillance for low-risk, localized PCa to a 16-week exercise intervention (home-based walking program; *n* = 26) or printed physical activity recommendations (control group; *n* = 25). Fasting blood samples were collected at baseline and after 16 weeks. Samples were analyzed for markers of insulinemia (insulin, C-peptide, adiponectin), inflammation (C-reactive protein (CRP)), and prostate-specific antigen (PSA). Biomarker changes over time and between arms were analyzed using linear mixed-effects models and intention-to-treat analysis.

**Results:**

22 (85%) intervention and 23 (92%) control participants, mean (SD) age of 63.6 (6.6) years, provided two blood samples. Average baseline biomarker values were within expected ranges. Analyses showed no changes within or differences in changes between intervention and control groups from baseline to 16 weeks for any biomarker (*p* > 0.05).

**Conclusions:**

We observed no changes in markers of insulinemia, inflammation, or PSA from a 16-week at-home walking intervention vs. control in individuals with low-risk PCa.

## Introduction

Prostate cancer (PCa) is the most commonly diagnosed cancer and the second leading cause of cancer death among men in the United States [1]. We and others have reported that physical activity is associated with a reduced risk of PCa progression and death [2–5]. For example, in an observational cohort of 2705 men with non-metastatic PCa, we previously found that men who engaged in weekly vigorous exercise had lower all-cause mortality (*p* < 0.001) and prostate cancer-specific mortality (PCSM) (*p* < 0.001) than men who engaged in an average of less than one hour per week (h/wk) of vigorous activity [2]. In a distinct cohort, we also reported that walking briskly for three h/wk or more (compared to walking at a leisurely pace for less than three h/wk) was associated with a 57% lower rate of PCa progression [3]. Other observational studies have corroborated these results, reporting that post-diagnosis physical activity is inversely associated with PCSM for men with localized, low-risk PCa [4, 6].

Two commonly cited mechanisms, insulin resistance and inflammation, are postulated to explain exercise’s influence on the modulation of cancer progression. High insulin levels and chronic or persistent inflammation have been tied to increased risk of multiple cancer types, including PCa [7, 8]. Poorer PCa survival outcomes have been associated with increased insulinemic markers in observational cohorts, with studies showing a 60–70% increased risk of PCSM for every standard deviation increase in triglyceride-glucose index values, a measure of insulin resistance, when the index was evaluated within ten years after PCa diagnosis [8, 9]. We previously reported that diet and lifestyle (e.g., body weight, physical activity per week) scores optimized to reflect hyperinsulinemia, insulin resistance, or inflammation were associated with an approximate 20–34% greater risk of PCa progression [10].

Exercise has been shown to improve adiposity and insulin sensitivity in various studies, including in the setting of cancer [2, 5, 11, 12].Most data on physical activity and PCa progression are from observational studies, and it remains unknown if *changing health habits after PCa diagnosis* influences progression or survival outcomes. There is evidence that exercise training can counteract the adverse side effects of androgen deprivation therapy (ADT) on body composition and metabolic markers in men with PCa.^[12,13]^Some studies have also shown that exercise can decrease prostate-specific antigen (PSA), a PCa tumor marker associated with PCa, and slow the rate of PSA increase, potentially indicating slower tumor progression [11, 14]. Less is known about how exercise training may affect insulin and inflammatory signaling, which may impact PCa progression in men with low-risk, localized PCa on active surveillance (AS). Previous studies have shown that exercise decreases anxiety around PCa diagnosis and have hypothesized that exercise may slow cancer progression and delay the need for treatment [15, 16]. Low-risk PCa, as seen in AS, may represent an opportunity to alter insulin and inflammatory cancer promotion before systemic disease causes further disruption to these pathways.

This study examined the effects of exercise training on blood-based biomarkers of insulin resistance and inflammation in patients with PCa opting for AS and whether exercise training impacted patients’ decisions to remain on AS. Insulin, C-peptide, adiponectin, and C-reactive protein (CRP) were selected to capture participants’ overall insulin sensitivity and inflammation profiles before and after the intervention, and serum PSA changes were investigated as a marker of tumor progression [11, 14]. Briefly, intervention studies have shown that lower serum insulin and C-peptide levels are associated with improved insulin plasma clearance and less insulin resistance. [17] Adiponectin has been associated with increased insulin sensitivity and decreased inflammation [11]. CRP is a widely used marker of inflammation that is increased in many cancer types, including PCa [18]. We also report adherence to AS, which was an a priori secondary outcome of the trial. Previous results from this trial suggest that participants in the exercise arm exhibited decreased fear of PCa recurrence, and previous studies hypothesized that exercise may slow PCa progression, so we investigated whether there were differences in AS durability based on exercise intervention [15, 16]. We hypothesized that a 16-week home-based aerobic exercise intervention (vs. control) would improve insulin sensitivity, inflammation, PSA doubling time (PSADT), and time on AS in men diagnosed with PCa on AS*.*

## Methods

This investigation leveraged data and specimens from the Active Surveillance Exercise (ASX) randomized controlled trial (RCT), a two-arm RCT conducted at the University of California, San Francisco (UCSF). The design and primary results of the study have been reported in more detail elsewhere.^16^ The UCSF Institutional Review Board approved all research, patients provided written informed consent, and CONSORT reporting guidelines were followed.

### Study population, screening procedures, and randomization

ASX trial participants were recruited between June 2016 and December 2021. All patients’ 24-month follow-ups were completed by February 2024. ASX enrolled men with biopsy-proven, non-metastatic clinical stage T2 or lower PCa who opted to undergo AS. Patients had to have no contraindications to vigorous exercise (as determined by the study physician and their primary care doctor), have a normal resting and exercise electrocardiogram (ECG), be able to complete a cardiopulmonary exercise test, and have low-to-moderate fitness based on age-specific peak oxygen uptake (VO_2peak_) cut points [16]. Participants were randomized to the exercise intervention arm or standard of care control arm in a 1:1 ratio.

### Intervention

Men in the intervention group were given a 16-week home-based walking program designed to increase cardiorespiratory fitness (CRF). Assigned exercise intensity steadily increased and varied from 45 to 80% VO_2peak_, as assessed by their heart rates during exercise, with session time ranging from 20 to 60 min. Participants initially exercised three days/week and increased to four days/week by week five. Participants were given and instructed to wear a Polar H10 heart rate monitor during exercise sessions, and heart rate results were recorded in the Polar app and reviewed by the exercise physiologist to ensure program adherence (as measured by heart rate data during exercise sessions) and safety. Additionally, intervention group participants were assigned an exercise physiologist with whom they had 10–15-min weekly calls to review completed sessions, reasons for missed sessions, and adverse events. Participants randomized to the control arm received printed materials with physical activity recommendations and the standard of care for PCa AS.

Additional details on the ASX trial methods and processes have been previously described by Van Blarigan et al. [16] The primary results indicated an improved VO_2peak_ for participants in the intervention vs. control arm without statistically significant changes in weight or waist circumference [16].

### Outcomes

At baseline and 16 weeks, participants provided fasting blood samples before CRF testing. This study assessed changes in circulating insulin, C-peptide, adiponectin, and CRP. All blood samples were collected via simple blood drawn after at least 8 h of fasting, separated into components (plasma, red blood cells, white blood cells), aliquoted, and stored at − 80 °C at UCSF. Frozen plasma samples were sent to Dr. Michael Pollak’s lab at McGill University, where they were analyzed for biomarkers using a quantitative sandwich enzyme immunoassay technique.

This study also evaluated the impact of the intervention on adherence to AS (defined as maintenance of AS without surgical, radiation, or chemotherapy intervention) at 12 and 24 months and explored clinical PSA measurements. Adherence to AS was defined as an a priori secondary outcome from the original trial. Clinical PSA measurements (a well-established biomarker for monitoring PCa progression on AS^19^) were monitored every 3–6 months via blood drawn as part of AS protocol. Adherence to AS was assessed via manual electronic health record (EHR) chart review for each patient at the conclusion of the study to determine whether, when, and what PCa treatment was performed.

PSA data were collected from the EHR using the value nearest to and directly before enrollment in the trial and the value nearest to and directly after the 16-week intervention period. PSA data were also collected for up to 24 months following the date of randomization to determine PSADT. PSA values were used to calculate PSADT for up to 24 months post-randomization, and participants were censored from analysis at the time of PCa treatment. PSADT was calculated as the natural log of 2 divided by the PSA slope (http://www.nomogram.org/).

### Statistical analyses

An intention-to-treat analysis and complete case analysis were performed. Biomarker values at baseline and 16 weeks for each study group were described using the median and lower and upper quartiles. We averaged our two log2-transformed biomarker measurements in our modeling but not in our graphics. We used linear mixed-effects models to examine post-intervention biomarker changes while controlling for baseline levels. Each model had fixed effects for group (intervention or control) and time (baseline or week 16), an interaction between group and time, and a random intercept for the participant; our scientific interest was in the interaction. Of the 51 participants enrolled in the trial, 50 participants had baseline biomarker data and 45 had 16-week biomarker data; thus, there were 45 pairs for two-timepoint analyses. We also used linear mixed-effects models to determine whether there was any association between change in fitness (estimated by VO_2peak_) and changes in the biomarkers’ values. Because PSA and adherence to AS data were gathered from the EHR, all 51 study participants were included in analyses involving these parameters. Differences in adherence to AS at 12 and 24 months and PSADT (binarized as > or <  = 24 months) were performed using chi-squared tests.

## Results

Table 1 shows the demographic and clinical characteristics of each study arm and the overall analytical population. No material differences were observed between the intervention and control groups. Five of the six participants excluded from this analysis due to missing blood samples had only one of the two blood draws. Of these, three patients had baseline biomarker readings within normal ranges, while two patients had slightly elevated C-peptide levels. No differences were observed between patients who provided two blood samples and those who did not, and those who did not provide blood samples either refused to provide both blood samples (*n* = 1), did not provide 16-week draw (*n* = 1), or withdrew (*n* = 4) from the study before providing samples at 16 weeks.

**Table 1:** Participant baseline demographic, clinical, and behavioral characteristics, overall and stratified by trial arm

Variable	Overall (*n* = 45)	Exercise arm (*n* = 22)	Standard of care arm (*n* = 23)
Sociodemographic characteristics			
Age, mean (SD), years	63.6 (6.6)	62.2 (5.3)	64.9 (7.6)
Race, No. (%)			
* African American / Black*	2 (4)	1 (5)	1 (4)
* American Indian / Alaskan Native*	1 (2)	0 (0)	1 (4)
* Asian*	2 (4)	1 (5)	1 (4)
* White*	38 (84)	19 (86)	19 (83)
* Other race*	2 (4)	2 (9)	0 (0)
Hispanic or Latino, No. (%)	5 (11)	2 (9)	3 (13)
Education, no. (%)			
* High school or less*	4 (89)	1 (5)	3 (13)
* 2- or 4-yr college*	17 (38)	11 (50)	6 (26)
* Graduate or professional school*	24 (53)	10 (45)	14 (61)
Occupational status, no. (%)			
* Full time*	26 (58)	12 (54)	14 (61)
* Part time*	3 (7)	2 (9)	1 (4)
* Looking for work*	1 (2)	1 (5)	0 (0)
* Retired*	15 (33)	7 (32)	8 (35)
Household income ($), no. (%)			
< *50,000*	1 (2)	1 (5)	1 (4)
* 50,000-* < *100,000*	9 (20)	5 (23)	4 (17)
* 100,00-* < *150,000*	5 (11)	2 (9)	3 (13)
* 150,000-* < *250,000*	10 (22)	5 (23)	5 (22)
> = *250,000*	17 (38)	9 (41)	8 (35)
Behavioral characteristics			
Smoking status, no. (%)			
* Current smoker*	1 (2)	1 (5)	0 (0)
* Former smoker*	10 (22)	4 (18)	6 (26)
* Never smoker*	34 (76)	17 (77)	17 (74)
Weight Training (m/wk), mean (SD)	56 (107)	61 (133)	52 (80)
Prostate cancer characteristics			
Clinical state, no. (%)			
*T1b*	1 (2)	1 (5)	0 (0)
*T1c*	25 (56)	14 (64)	11 (48)
*T2a*	15 (33)	5 (23)	10 (43)
*T2b*	1 (2)	0 (0)	1 (4)
*T2c*	3 (7)	2 (9)	1 (4)
Gleason grade, no. (%)			
*3* + *3*	31 (69)	17 (77)	14 (61)
*3* + *4*	14 (31)	5 (23)	9 (39)
PSA level at diagnosis (ng/ml), mean (SD)	5.4 (2.4)	6.2 (3.0)	4.7 (1.4)
PSA density (ng/ml/cc), mean (SD)	0.13 (0.05)	0.14 (0.04)	0.12 (0.05)
Time on active surveillance before study (mo), mean (SD)	30 (37)	21 (33)	38 (40)

*cc* cubic centimeters, *h/wk* hours per week, *IQR* inner quartile range, *ml* milliliters, *mo* months, *m/wk* minutes per week, *ng* nanograms, *no* number, *PSA* prostate-specific antigen, *SD* standard deviation.

**Fig. 1:**
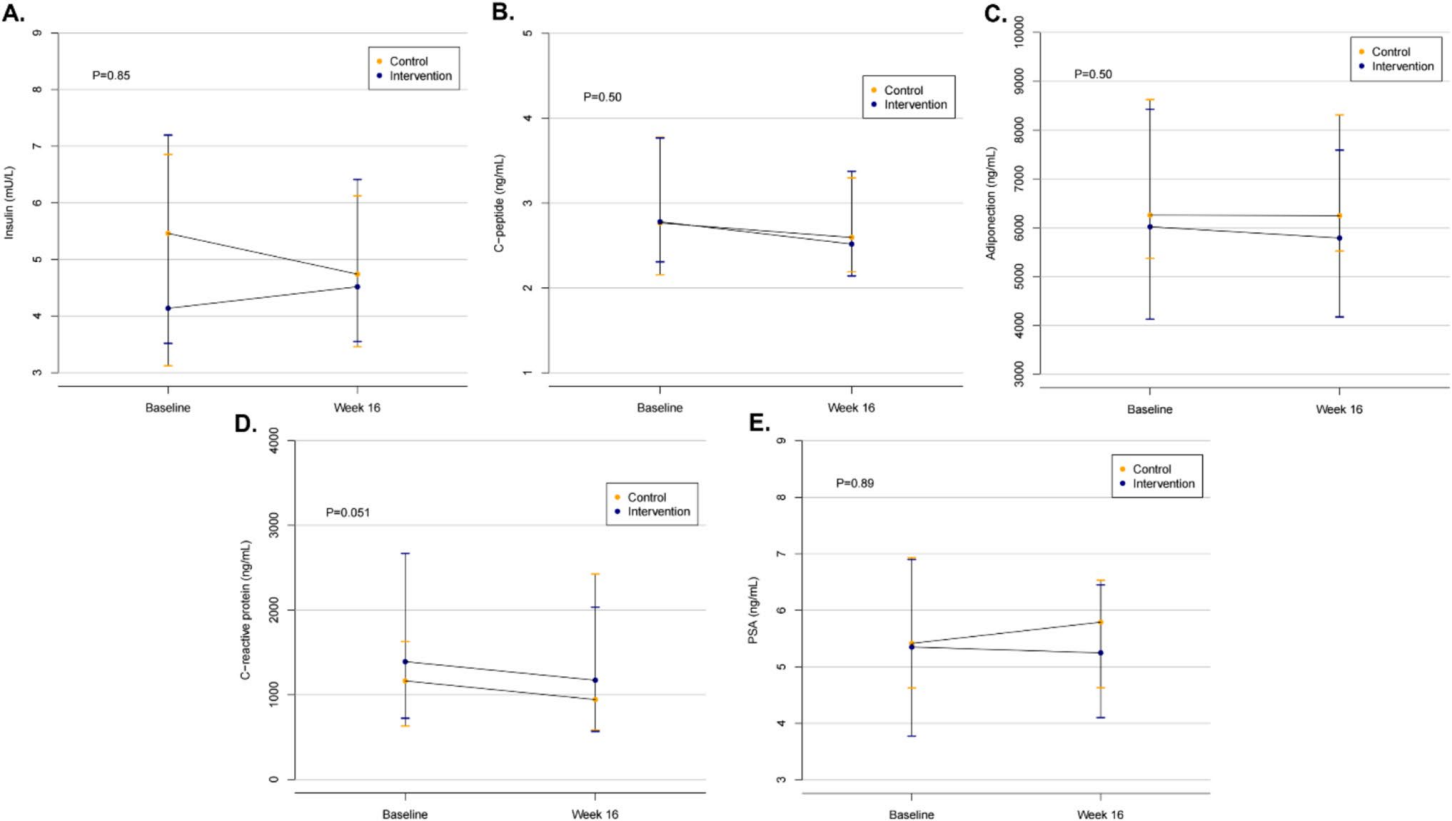
Effect of a 16-week home-based walking program versus usual care on blood-based insulin sensitivity **A**. fasting insulin, **B**. C-peptide, **C**. adiponectin levels), inflammation **D**. C-reactive protein levels), and plasma PSA **E**. in 45 men on active surveillance for prostate cancer. *p*- values for the difference in change between control and exercise intervention group over the program duration are given on each graph. Dots indicate mean while bars indicate 95% confidence intervals. Abbreviations: PSA prostate-specific antigen. References values: Insulin, serum (fasting): < 20 mU/L [24]; C-peptide, serum: 0.8–3.1 ng/mL [24]; Adiponectin: 3–30 μg/ml [23]; C-reactive protein, serum: ≤ 0.8 mg/dL [24]; Prostate-specific antigen, serum: ng/mL; no specific normal or abnormal level [24]

Figure 1 provides insulin, C-peptide, adiponectin, CRP, and PSA values for each study arm at baseline and 16 weeks. No differences in changes for any of these biomarkers were seen between the intervention and control arms (*p* > 0.05). An analysis of changes within groups was also performed, showing that neither group demonstrated significant alterations in any biomarkers from baseline to 16 weeks (*p* > 0.05). When investigating associations between changes in VO_2peak_ and changes in the biomarkers from baseline to 16 weeks, no relationships were observed (*p* > 0.05).

Among participants who had sufficient PSA data (*n* = 40 who had both pre- and at least two post-intervention values available), no differences in PSADT (using a threshold of < 24 months) were observed between groups. Four out of 22 (18%) participants in the control arm and eight out of 18 (44%) in the intervention arm had PSADT < 24 months (*p* = 0.56). Investigating adherence to AS, seven (14%) (three in the control arm and four in the intervention arm) participants reported treatment by 12 months, and 16 (31%) (nine in the control arm and seven in the intervention arm) reported treatment by 24 months. By 24 months following trial enrollment, nine had undergone surgery, six had received radiation therapy, and one had undergone focal cryoablation. There were no differences in AS maintenance between groups at 12 or 24 months (*p* = 0.99 and *p* = 0.69, respectively).

## Discussion

This study evaluated the effects of a 16-week home-based walking program on biomarkers of insulinemia and inflammation and PSA among 45 men from the ASX trial [16]. We did not observe any differences in change between groups or any changes within groups for insulin, C-peptide, adiponectin, CRP, or PSA levels, the means of which were within normal ranges (except for PSA) throughout the study [20–24].

These results are different from those of prior RCTs that have investigated how exercise after a diagnosis of cancer impacts cancer outcomes, insulinemia, and inflammation. One meta-analysis of 26 RCTs varying from durations of 8–104 weeks and offering structured exercise interventions for cancer survivors investigated exercise’s effect on cytokine levels, including CRP and TNF-α, and showed decreased pro-inflammatory markers with exercise across all cancer types [18]. The study also showed that CRP levels are associated with cancer-specific mortality in more advanced disease [18]. Combined aerobic and resistance training had the greatest effect on inflammation, and prostate and breast cancer patients were most responsive to the interventions [18]. Notably, our study only featured aerobic exercise. One two-arm RCT investigating the effects of 8 weeks of supervised exercise vs. control on inflammatory markers in a population of patients undergoing radiotherapy for prostate cancer (*N* ~ 72) showed a decrease in overall cytokine levels with exercise [25]. They also reported that exercise during and ten months after radiotherapy vs. control significantly lowered body mass index (BMI), waist-to-hip ratio, PSA, and IL-6 levels compared to usual care [25]. A separate two-arm RCT of 19 patients on AS or with biochemical recurrence of PCa following radical prostatectomy indicated that a 2-year home-based endurance training intervention slowed PSADT, lowered BMI, and improved adiponectin but did not alter insulin sensitivity or inflammatory markers, indicating that even longer interventions may or may not impact insulin sensitivity and inflammatory biomarkers [11].

The Exercise During Active Surveillance for Prostate Cancer (ERASE) RCT was an exercise-only two-armed RCT with 52 participants, 26 in each arm, assigned to a supervised 12-week high-intensity interval training (HIIT) training program or usual care. Results showed increased VO_2peak_ following the intervention, decreased PSA levels, decreased PSA velocity, decreased growth of PCa cell lines, and decreased insulin compared to the control arm; however, they did not see any differences in adiponectin and did not investigate C-peptide [14, 26]. The ERASE trial was similar to ASX in duration (12 weeks for ERASE versus 16 weeks for ASX), showed a smaller average VO_2peak_ change improvement (1.6 ml/kg/min for ERASE versus 3.7 ml/kg/min for ASX), and used a similar population, but ERASE used a supervised exercise protocol centered around HIIT training rather than the unsupervised home walking program used by ASX [14, 16]. Our per-protocol analyses did not identify any associations between changes in VO_2peak_ and alterations in the blood-based biomarkers, and these associations were not investigated in ERASE. Compared to supervised HIIT training, ASX is unique because the program was home-based and remotely monitored, representing a more accessible and less resource-intensive form of exercise. The ASX trial also showed decreased anxiety about PCa progression, but no differences in AS maintenance were observed between groups [16]. To the best of our knowledge, no other groups have investigated adherence to PCa AS in the setting of post-diagnostic exercise interventions, but previous studies hypothesized that exercise may slow cancer progression and delay the need for treatment [15, 16].

While some of these trials demonstrated decreases in insulinemia, inflammation, and PSA with exercise, there have been inconsistencies in which biomarkers are improved and by how much. Some examples of differences between other studies and ours included radiotherapy or radical prostatectomy versus AS for PCa, treatment with ADT, different grades of disease, supervised versus unsupervised exercise, and duration of intervention. More aggressive disease and treatment in these study participants might have contributed to increasingly inflammatory environments, making them more amenable to change via exercise intervention. Other possible explanations for the null findings observed in ASX compared to other RCTs could be related to anthropometric differences in the study population. For example, in ASX, the participants’ average baseline levels of our evaluated biomarkers were within normal healthy ranges and thus could have been less likely to be affected by the intervention, whereas some other studies selected men on ADT and those who did not engage in vigorous exercise [12–14]. While participants were selected based on having lower CRF, they were not necessarily overweight or obese and did not experience a marked reduction in weight during the trial [16].

The mechanism by which exercise impacts early-stage PCa, if not via insulin signaling and inflammation, remains ambiguous. New research has shown decreased rates of PCa incidence for men who exercise and have higher CRF [27]. This may be because CRF adaptations involve both central (cardiac output) and peripheral (muscle oxidative capacity) mechanisms, which may not always align directly with pathways on insulin regulation [27]. Other studies have also shown that exercise has a positive effect on PSA levels for men already diagnosed with PCa who are on AS [28]. Several of the above studies also indicate that changes in fitness after exercise are linked to positive effects on PSA [11, 14, 25]. The links between CRF and PCa outcomes have yet to be fully explained and warrant additional investigation to determine the mechanisms by which CRF impacts PCa.

Strengths of this study include its high exercise adherence rates, feasible implementation, broad generalizability to men with low-risk PCa, follow-up time in looking at AS adherence, and in-depth assessment of insulinemic biomarkers. However, this study had several limitations. The analysis population was small (*n* = 45). The 16-week duration of the exercise intervention may have been insufficient to improve insulinemic and inflammatory markers, particularly given that our participant’s biomarkers started in the normal value reference ranges. [29, 30] Inclusion of a wider array of insulin resistance and inflammation biomarkers, such as the homeostatic model assessment of insulin resistance, TNF-α, and interleukins, might have detected changes that our biomarkers did not. A more intense intervention, one including weight or resistance training, or longer duration may also have been needed to observe changes in these indicators, especially in relatively healthy men.

In conclusion, while the ASX trial previously observed that fitness and aspects of quality of life were improved via a remotely monitored exercise program in the setting of men with PCa on AS, the intervention did not produce detectable changes in blood-based biomarkers of inflammation and insulinemia [16]. Moreover, although anxiety around PCa progression was eased, there were no differences in adherence to AS based on treatment group. Further research is needed to understand if increases in exercise or improvements in CRF affect PCa progression, and, if so, by what mechanisms.

## Data Availability

No datasets were generated or analysed during the current study.

## References

[1] Siegel RL, Miller KD, Fuchs HE, Jemal A (2021) Cancer statistics, 2021. CA Cancer J Clin 71(1):7–33. 10.3322/caac.21654 33433946 10.3322/caac.21654

[2] Kenfield SA, Stampfer MJ, Giovannucci E, Chan JM (2011) Physical activity and survival after prostate cancer diagnosis in the Health Professionals Follow-Up Study. J Clin Oncol. 10.1200/JCO.2010.31.5226 21205749 10.1200/JCO.2010.31.5226 PMC3056656

[3] Richman EL, Kenfield SA, Stampfer MJ, Paciorek A, Carroll PR, Chan JM (2011) Physical activity after diagnosis and risk of prostate cancer progression: data from the cancer of the prostate strategic urologic research endeavor. Cancer Res 71(11):3889–3895. 10.1158/0008-5472.CAN-10-3932 21610110 10.1158/0008-5472.CAN-10-3932 PMC3107352

[4] Friedenreich CM, Wang Q, Neilson HK, Kopciuk KA, McGregor SE, Courneya KS (2016) Physical activity and survival after prostate cancer. Eur Urol 70(4):576–585. 10.1016/j.eururo.2015.12.032 26774959 10.1016/j.eururo.2015.12.032

[5] Hojman P, Gehl J, Christensen JF, Pedersen BK (2018) Molecular mechanisms linking exercise to cancer prevention and treatment. Cell Metab 27(1):10–21. 10.1016/j.cmet.2017.09.015 29056514 10.1016/j.cmet.2017.09.015

[6] Wang Y, Jacobs EJ, Gapstur SM et al (2017) Recreational physical activity in relation to prostate cancer-specific mortality among men with nonmetastatic prostate cancer. Eur Urol 72(6):931–939. 10.1016/j.eururo.2017.06.037 28711382 10.1016/j.eururo.2017.06.037

[7] Jin Q, Shi N, Lee DH et al (2023) Hyperinsulinemic and pro-inflammatory dietary patterns and metabolomic profiles are associated with increased risk of total and site-specific cancers among postmenopausal women. Cancers 15(6):1756. 10.3390/cancers15061756 36980642 10.3390/cancers15061756 PMC10046106

[8] Sfanos KS, De Marzo AM (2012) Prostate cancer and inflammation: the evidence. Histopathology 60(1):199–215. 10.1111/j.1365-2559.2011.04033.x 22212087 10.1111/j.1365-2559.2011.04033.x PMC4029103

[9] Jochems SHJ, Fritz J, Häggström C, Stattin P, Stocks T (2023) Prediagnostic markers of insulin resistance and prostate cancer risk and death: a pooled study. Cancer Med 12(12):13732–13744. 10.1002/cam4.6004 37102250 10.1002/cam4.6004 PMC10315749

[10] Langlais CS, Graff RE, Van Blarigan EL et al (2022) Postdiagnostic inflammatory, hyperinsulinemic, and insulin-resistant diets and lifestyles and the risk of prostate cancer progression and mortality. Cancer Epidemiol Biomarkers Prev 31(9):1760–1768. 10.1158/1055-9965.EPI-22-0147 35767977 10.1158/1055-9965.EPI-22-0147 PMC9444922

[11] Hvid T, Lindegaard B, Winding K et al (2016) Effect of a 2-year home-based endurance training intervention on physiological function and PSA doubling time in prostate cancer patients. Cancer Causes Control 27(2):165–174. 10.1007/s10552-015-0694-1 26573844 10.1007/s10552-015-0694-1

[12] Hvid T, Winding K, Rinnov A et al (2013) Endurance training improves insulin sensitivity and body composition in prostate cancer patients treated with androgen deprivation therapy. Endocr Relat Cancer 20(5):621–632. 10.1530/ERC-12-0393 23744766 10.1530/ERC-12-0393

[13] Galvão DA, Taaffe DR, Spry N, Joseph D, Newton RU (2010) Combined resistance and aerobic exercise program reverses muscle loss in men undergoing androgen suppression therapy for prostate cancer without bone metastases: a randomized controlled trial. J Clin Oncol Off J Am Soc Clin Oncol 28(2):340–347. 10.1200/JCO.2009.23.2488 10.1200/JCO.2009.23.2488 19949016

[14] Kang DW, Fairey AS, Boulé NG, Field CJ, Wharton SA, Courneya KS (2021) Effects of exercise on cardiorespiratory fitness and biochemical progression in men with localized prostate cancer under active surveillance: the ERASE randomized clinical trial. JAMA Oncol 7(10):1487–1495. 10.1001/jamaoncol.2021.3067 34410322 10.1001/jamaoncol.2021.3067 PMC8377605

[15] Brassetti A, Cacciatore L, Bove AM et al (2024) The impact of physical activity on the outcomes of active surveillance in prostate cancer patients: a scoping review. Cancers 16(3):630. 10.3390/cancers16030630 38339381 10.3390/cancers16030630 PMC10854832

[16] Blarigan ELV, Kenfield SA, Olshen A et al (2024) Effect of a Home-based Walking Intervention on Cardiopulmonary Fitness and Quality of Life Among Men with Prostate Cancer on Active Surveillance: The Active Surveillance Exercise Randomized Controlled Trial. Eur Urol Oncol. 10.1016/j.euo.2023.10.012 37907387 10.1016/j.euo.2023.10.012 PMC12535774

[17] Wirth A, Diehm C, Mayer H et al (1981) Plasma C-peptide and insulin in trained and untrained subjects. J Appl Physiol 50(1):71–77. 10.1152/jappl.1981.50.1.71 7009528 10.1152/jappl.1981.50.1.71

[18] Khosravi N, Stoner L, Farajivafa V, Hanson ED (2019) Exercise training, circulating cytokine levels and immune function in cancer survivors: a meta-analysis. Brain Behav Immun 81:92–104. 10.1016/j.bbi.2019.08.187 31454519 10.1016/j.bbi.2019.08.187

[19] Cooperberg MR, Meeks W, Fang R, Gaylis FD, Catalona WJ, Makarov DV (2023) Time trends and variation in the use of active surveillance for management of low-risk prostate cancer in the US. JAMA Netw Open 6(3):e231439. 10.1001/jamanetworkopen.2023.1439 36862409 10.1001/jamanetworkopen.2023.1439 PMC9982696

[20] Woloshin S, Schwartz LM (2005) Distribution of C-reactive protein values in the United States. N Engl J Med 352(15):1611–1613. 10.1056/NEJM200504143521525 15829550 10.1056/NEJM200504143521525

[21] Williams Textbook of Endocrinology - ClinicalKey.https://www.clinicalkey.com/#!/browse/book/3-s2.0-C20210020964 Accessed 10, Sept 2024

[22] Yosten GLC, Maric-Bilkan C, Luppi P, Wahren J (2014) Physiological effects and therapeutic potential of proinsulin C-peptide. Am J Physiol Endocrinol Metab 307(11):E955–E968. 10.1152/ajpendo.00130.2014 25249503 10.1152/ajpendo.00130.2014 PMC4254984

[23] Ramakrishnan N, Auger K, Rahimi N, Jialal I. (2024) Biochemistry, Adiponectin. In: *StatPearls*. StatPearls Publishing; http://www.ncbi.nlm.nih.gov/books/NBK537041/. Accessed 10, Sept, 2024. 30725726

[24] ABIM Laboratory Test Reference Ranges – (2024) Published online Jan 2024. https://www.abim.org/Media/bfijryql/laboratory-reference-ranges.pdf. Accessed 7, Oct 2024

[25] Hojan K, Kwiatkowska-Borowczyk E, Leporowska E, Milecki P. (2017) Inflammation, cardiometabolic markers and functional changes in a randomized controlled trial of a 12-month exercise program for prostate cancer men. *Pol Arch Intern Med*. Published online 2017 10.20452/pamw.3888 10.20452/pamw.3888 28075422

[26] Kang DW, Field CJ, Patel D et al (2024) Effects of high-intensity interval training on cardiometabolic biomarkers in patients with prostate cancer undergoing active surveillance: a randomized controlled trial. Prostate Cancer Prostatic Dis. 10.1038/s41391-024-00867-3 39009705 10.1038/s41391-024-00867-3

[27] Bolam KA, Bojsen-Møller E, Wallin P et al (2024) Association between change in cardiorespiratory fitness and prostate cancer incidence and mortality in 57 652 Swedish men. Br J Sports Med 58(7):366–372. 10.1136/bjsports-2023-107007 38290798 10.1136/bjsports-2023-107007 PMC10982617

[28] Lee DJ, Byeon JY, Park DH et al (2024) Effects of exercise during active surveillance for prostate cancer: a systematic review and meta-analysis. Support Care Cancer 32(7):406. 10.1007/s00520-024-08606-z 38833183 10.1007/s00520-024-08606-z

[29] Zheng G, Qiu P, Xia R et al (2019) Effect of Aerobic Exercise on Inflammatory Markers in Healthy Middle-Aged and Older Adults: A Systematic Review and Meta-Analysis of Randomized Controlled Trials. Front Aging Neurosci. 10.3389/fnagi.2019.00098 31080412 10.3389/fnagi.2019.00098 PMC6497785

[30] Bird SR, Hawley JA (2017) Update on the effects of physical activity on insulin sensitivity in humans. BMJ Open Sport Exerc Med 2(1):e000143. 10.1136/bmjsem-2016-000143 28879026 10.1136/bmjsem-2016-000143 PMC5569266

